# The Moderating Role of Coping Mechanisms and Being an e-Sport Player Between Psychiatric Symptoms and Gaming Disorder: Online Survey

**DOI:** 10.2196/21115

**Published:** 2021-03-23

**Authors:** Fanni Bányai, Ágnes Zsila, Gyöngyi Kökönyei, Mark D Griffiths, Zsolt Demetrovics, Orsolya Király

**Affiliations:** 1 Institute of Psychology ELTE Eötvös Loránd University Budapest Hungary; 2 Doctoral School of Psychology ELTE Eötvös Loránd University Budapest Hungary; 3 Institute of Psychology Pázmány Péter Catholic University Budapest Hungary; 4 SE-NAP2 Genetic Brain Imaging Migraine Research Group Hungarian Academy of Sciences Semmelweis University Budapest Hungary; 5 Department of Pharmacodynamics Faculty of Pharmacy Semmelweis University Budapest Hungary; 6 International Gaming Research Unit Psychology Department Nottingham Trent University Nottingham United Kingdom; 7 Centre of Excellence in Responsible Gaming University of Gibraltar Gibraltar Gibraltar

**Keywords:** gaming disorder, esports, professional gaming, video games, coping skills, psychiatric symptoms, psychiatry, mental health, gaming

## Abstract

**Background:**

The emerging popularity of playing video games (*gaming*) as a hobby and as a professional sport raises awareness about both the benefits and possible downsides of the activity. Although a healthy and passionate hobby for most, a minority of gamers experience addiction-like symptoms and are considered to have gaming disorder (GD). GD has previously been found to be related to aversive conditions, such as depression or anxiety, as well as putatively maladaptive coping strategies.

**Objective:**

The aim of this study is twofold: to explore the moderating effect of different coping strategies and type of video game usage (professional [e-sport] or recreational) on the relationship between psychiatric symptoms and GD.

**Methods:**

A sample of 3476 gamers (n=3133, 90.13% males; mean age 23.20, SD 6.48 years) was recruited via the website and social networking site of the most popular gaming magazine in Hungary (*GameStar*).

**Results:**

The main effect of psychiatric symptoms was moderate to large in all models, whereas the moderation effects were significant (*P*<.001) for 4 out of 8 coping strategies (ie, self-blame/self-distraction, denial, emotional/social support, and active coping). However, the explained variance of the models only increased negligibly (from 0.3% to 0.5%) owing to the moderation effect. The direction of the moderations was as expected (ie, putatively maladaptive strategies were associated with more GD symptoms when the level of psychiatric symptoms was high, while putatively adaptive strategies were associated with less). Furthermore, no considerable moderation effect of the player type (recreational vs professional players) was found on the association between psychiatric symptoms and GD (*β*=.04; *P*=.02; 0.1% change in the explained variance).

**Conclusions:**

Future studies should be designed to better understand coping-related mechanisms in the context of video gaming and GD.

## Introduction

Video game playing (*gaming*) has become one of the most popular leisure activities globally irrespective of age and gender [1]. Its great popularity has led to the phenomenon of *e-sports*, which refers to professional competitive gaming where teams or individuals compete against each other in a video game [2-5]. There are now organized and sanctioned e-sport competitions worldwide that are hosted by sponsors, featuring live sports commentary. These are watched by large-scale audiences (both at scene and via online streaming platforms such as *Twitch.tv, YouTube, etc*), and there are big money prizes for the winners [6].

Although the overwhelming majority of gamers globally play in a healthy manner, a small minority experience addiction-like symptoms accompanied by marked psychological distress and significant impairment in personal, family, social, educational, occupational, and/or other important areas of functioning [7]. The severity of the problem is acknowledged by the inclusion of internet gaming disorder (IGD) in Section 3 (*Emerging Measures and Models*) of the fifth edition of the Diagnostic and Statistical Manual of Mental Disorders in 2013 as a condition warranting further research [8] and by the inclusion of gaming disorder (GD) in the 11th revised edition of the International Classification of Diseases in 2019 as an official diagnosis [9]. Furthermore, there is a wide variety of terms used for problematic or addictive video gaming. This paper uses the term *GD* given that it is the official term proposed by the World Health Organization.

Similar to substance use or alcohol use disorders, GD has been found to be related to psychiatric symptoms such as depression and anxiety according to numerous epidemiological survey studies [10-12]. One important issue regarding these findings is whether there are factors that moderate these associations. More specifically, the negative effects of emotional or psychiatric distress on an individual’s life may depend on the individual’s ability to cope with it [13]. Coping can be defined as the cognitive and behavioral responses of individuals in an attempt to manage stressful situations and emotions associated with them [14]. Considering that the context and goals of the individual strongly determine the effectiveness of the strategies [15,16], the adaptive-maladaptive classification could arguably be criticized. However, some strategies may be labeled as putatively maladaptive if they are associated with poor outcomes, especially in the long term. For instance, dispositional rumination—defined as the tendency to dwell on distress-related thoughts passively and repetitively [17]—and avoidance are consistently associated with psychopathology [18]. Similarly, some strategies might be considered as putatively adaptive since they are generally associated with good adjustment. For instance, acceptance of mental experiences is related to better psychological health [19].

Several studies have investigated the association between coping strategies and GD. According to these findings, GD is associated with putatively maladaptive or dysfunctional coping styles [20], such as denial, behavioral disengagement [21], media-related coping, self-distraction, self-blame [22-24], catastrophizing, or rumination [25]. In addition, putatively adaptive coping styles such as active coping, positive reframing, and positive reappraisal have been applied less frequently in the case of gamers at risk of GD or have been negatively related to GD [22,25]. Effect sizes range from weak to strong in the case of putatively maladaptive strategies and weak to moderate in the case of putatively adaptive coping styles.

Furthermore, several studies have tested more complex models, in which coping styles have been assumed to mediate between psychiatric symptoms or stress and GD. According to such models, higher rates of stress or specific psychiatric problems such as depression have been associated with or predicted the use of dysfunctional coping styles (eg, avoidance or media-focused coping). This, in turn, has been associated with (or predicted higher rates of) GD or general problematic internet use. Effect sizes for the psychiatric symptoms or coping style associations have been moderate or moderate-to-strong, whereas those for coping style or GD associations have been weak or weak to moderate [26-28].

Although it is plausible to think that increased psychiatric symptoms (eg, depression) may increase the risk of using dysfunctional coping strategies [29], it is also plausible to assume that dispositional coping styles may act as moderators between symptoms and GD. This means that they can influence the association between psychiatric symptoms and GD. It is logical to hypothesize that among individuals who frequently use putatively maladaptive or dysfunctional coping styles when encountering stressful situations in their lives, the relationship between psychiatric symptoms and GD will be stronger than among those who use putatively adaptive coping strategies in general. Findings reporting that escapism (ie, playing videogames to avoid problems and difficulties) is the motive most consistently related to GD [30-32] supports this hypothesis. Therefore, instead of mediation models, this study aims to test whether coping styles (both putatively adaptive and maladaptive) moderate the psychiatric symptoms or GD relationship in the aforementioned way.

The second aim of this study is to test whether player type (recreational vs e-sport players) moderated the association between psychiatric symptoms and GD. The large amount of time and energy that e-sport players spend training to improve their gaming skills and be successful in competitions raises the question of whether e-sport players may be at a higher risk of developing GD than recreational gamers [33]. To date, very few studies have investigated this risk, even though it affects a high number of aspiring e-sport players globally. According to a few previous studies, e-sport players do not report considerably higher GD scores than recreational gamers, and GD-related mechanisms also appear to be similar among e-sport players and highly engaged recreational players [30,31,34]. Therefore, a second assumption was that e-sport players will not significantly differ from highly engaged recreational players in the psychiatric symptoms or GD association.

## Methods

### Participants and Procedure

Participants were recruited via the website and social networking site (ie, *Facebook*) of the most popular gaming magazine in Hungary (*GameStar*). Data were collected using a web-based questionnaire that focused on both healthy and problematic (ie, addictive) use of video games. Participation was voluntary and anonymous. Gamers younger than 18 years (14-17 years of age) were allowed to participate in the survey after providing parental consent to participate. Two shopping vouchers (60,000 Hungarian forint, approximately US $260 each) were used as incentives and raffled among the gamers who participated in the survey. A contact email address was asked by the participants who joined the raffle. The email addresses were used only to inform the winners, and all contacts were deleted afterward.

A total of 7815 participants participated in the survey. According to the aim of this study, participants who provided data for all study-relevant variables (ie, psychiatric symptoms, coping strategies, and symptoms of GD) were included in the data analysis. Consequently, the final sample comprised 3476 gamers (n=3133, 90.13% males; mean age 23.20, SD 6.48 years). The study was approved by the Institutional Review Board of the research team’s university and was conducted in accordance with the Declaration of Helsinki.

### Measures

#### Sociodemographic Variables

Major sociodemographic data were collected, including age, gender, the number of years spent in education and working, and marital status.

#### Variables Relating to Video Game Use

Data related to general video game usage were also collected. Participants were asked to report their approximate game time on average hours/weekday and average hours/weekend day. The approximate game time hours/day was calculated as (5 × week day + 2 × weekend day) / 7. The average gaming time hours/week was calculated as 5 × week day + 2 × weekend day. To identify e-sport gamers and recreational gamers in the sample, participants were asked to indicate the types of competitions (ie, online or offline via local area network competitions) and the frequency of e-sport events they attended in the previous year (response options: “I did not participate in such competitions in the past year”; “1-2 times in the past year”; “3-5 times in the past year”; “6-11 times in the past year”; “several times a month”; and “weekly or more frequently”). Following the classification method of Bányai et al [34] and taking into consideration the theoretical concept [2,3,6] and the methods of how e-sport tournaments are organized, gamers who participated in e-sport tournaments at least 6-11 times in the previous year were defined as “e-sport gamers.” Gamers who participated in such tournaments only 5 times or fewer in the previous year were defined as “recreational gamers.”

#### Coping Strategies

Coping strategies were assessed using the Brief COPE scale [35]. The Brief COPE is a self-report scale assessing 14 different coping strategies (ie, self-distraction, active coping, denial, substance use, use of emotional support, use of instrumental support, behavioral disengagement, venting, positive reframing, planning, humor, acceptance, religion, and self-blame). Each coping strategy is represented by 2 items that are rated on a 4-point Likert scale (ranging from 1=“I haven’t been doing this at all” to 4=“I've been doing this a lot”). Several factors in the Brief COPE scale showed poor internal consistency in this study: self-distraction (*α*=.55), venting (*α*=.58), behavioral disengagement (*α*=.61), acceptance (*α*=.66), and planning (*α*=.69). Previous studies, which also found that the original factors of the Brief COPE questionnaire had low internal consistencies, explored alternative factor structures that yielded similar coping strategies but with better psychometric properties [36-38]. Following this conceptual framework, an exploratory factor analysis (EFA) was conducted to find an alternative factor structure for the Brief COPE. A total of 8 factors were identified according to the EFA, including emotional/social support, active coping, self-blame/self-distraction, humor, substance use, denial, religion, and acceptance. According to the aforementioned broad categorization, emotional/social support, active coping, humor, religion, and acceptance were considered to be putatively adaptive coping strategies, whereas self-blame/self-distraction, substance use, and denial were considered to be putatively maladaptive or dysfunctional coping strategies. The newly reconstructed factors showed better internal consistencies ranging between 0.78 and 0.92, except for the acceptance (*α*=.66) and self-blame/self-distraction factors (*α*=.68), which had *α* values below the .70 threshold (Tables 1 and 2).

**Table 1:** Factors obtained using exploratory factor analysis with Promax rotation.

Items of Brief COPE	1	2	3	4	5	6	7	8
“I’ve been getting help and advice from other people.” (COPE 10)	0.88	—^a^	—	—	—	—	—	—
“I've been getting emotional support from others.” (COPE 5)	0.85	—	—	—	—	—	—	—
“I've been getting comfort and understanding from someone.” (COPE 15)	0.85	—	—	—	—	—	—	—
“I’ve been trying to get advice or help from other people about what to do.” (COPE 23)	0.79	—	—	—	—	—	—	—
“I've been saying things to let my unpleasant feelings escape.” (COPE 9)	0.63	—	—	—	—	—	—	—
“I've been taking action to try to make the situation better.” (COPE 7)	—	0.82	—	—	—	—	—	—
“I've been concentrating my efforts on doing something about the situation I'm in.” (COPE 2)	—	0.82	—	—	—	—	—	—
“I've been trying to come up with a strategy about what to do.” (COPE 14)	—	0.79	—	—	—	—	—	—
“I've been thinking hard about what steps to take.” (COPE 25)	—	0.69	—	—	—	—	—	—
“I’ve been criticizing myself.” (COPE 13)	—	—	0.86	—	—	—	—	—
“I’ve been blaming myself for things that happened.” (COPE 26)	—	—	0.85	—	—	—	—	—
“I've been turning to work or other activities to take my mind off things.” (COPE 1)	—	—	0.58	—	—	—	—	—
“I've been making fun of the situation.” (COPE 28)	—	—	—	0.96	—	—	—	—
“I've been making jokes about it.” (COPE 18)	—	—	—	0.96	—	—	—	—
“I've been using alcohol or other drugs to make myself feel better.” (COPE 4)	—	—	—	—	0.95	—	—	—
“I've been using alcohol or other drugs to help me get through it.” (COPE 11)	—	—	—	—	0.95	—	—	—
“I've been saying to myself ‘this isn't real’.” (COPE 3)	—	—	—	—	—	0.90	—	—
“I've been refusing to believe that it has happened.” (COPE 8)	—	—	—	—	—	0.89	—	—
“I've been praying or meditating.” (COPE 27)	—	—	—	—	—	—	0.90	—
“I've been trying to find comfort in my religion or spiritual beliefs.” (COPE 22)	—	—	—	—	—	—	0.90	—
“I've been accepting the reality of the fact that it has happened.” (COPE 20)	—	—	—	—	—	—	—	0.87
“I've been learning to live with it.” (COPE 24)	—	—	—	—	—	—	—	0.86

^a^Factor loadings <0.10.

**Table 2:** Factors names and Cronbach alphas obtained using exploratory factor analysis with Promax rotation.

Factor names	Cronbach alpha
Emotional/social support	.86
Active coping	.79
Self-blame/ self-distraction	.68
Humor	.92
Substance use	.92
Denial	.78
Religion	.78
Acceptance	.66

#### Psychiatric Symptoms

Psychiatric symptoms were assessed using the Hungarian version of the Brief Symptom Inventory (BSI [39,40]). The scale comprises 53 items on a 5-point Likert scale (from *not at all*=0 to *extremely*=4), assessing 9 symptoms (ie, somatization, obsession-compulsion, interpersonal sensitivity, depression, anxiety, hostility, phobic anxiety, paranoid ideation, and psychoticism). In this study, 3 subscales of the BSI were used: depression (6 items), anxiety (6 items), and psychoticism (5 items). From the 3 BSI subscales, a summarized index named *Psychiatric Symptoms* was calculated to determine the intensity of general distress, which showed a strong relationship with GD in previous studies [11,30,31,34]. The *Psychiatric Symptoms* index with its respective 17 items showed good internal consistency in this study. Cronbach alpha was .93.

#### Gaming Disorder

The symptoms of GD were assessed using the Hungarian version of the 10-Item Internet Gaming Disorder Test (IGDT-10 [41]). The IGDT-10 was developed to assess the 9 criteria of IGD as proposed in the DSM-5. Each item of the IGDT-10 assesses 1 DSM-5 criterion, except for the final criterion (eg, “jeopardy or losing a significant relationship, job, or educational or career opportunity because of participation in internet games”), which was operationalized via 2 items to avoid double-barreled questioning. The IGDT-10 has 3 response options (*never*=0, *sometimes*=1, and *often*=2). To follow the dichotomous structure of the DSM-5, response options were recoded in the following way: *never* and *sometimes* options were recoded as *no* (0), while *often* responses as *yes* (1). The ninth and 10th items were recoded into a single item (ie, if any of the 2 original items had an “often” response, the new item was coded as “yes”) to resemble the original structure of the IGD. Composite reliability of the IGDT-10 was 0.88. Following the ICD-11 [9] classification of GD, the IGDT-10 scores specified in this study are used as an indicator of GD.

### Statistical Analysis

Data analysis was conducted using SPSS version 22.0 [42] with the PROCESS modeling tool version 2.16.3 [43]. EFA was conducted with principal component analysis with Promax rotation to explore the alternative factor structure of Brief COPE [44]. In the moderation models, the variable of psychiatric symptoms was the independent variable, whereas GD was the outcome variable. Coping strategies and player type (ie, recreational players or professional e-sport players) were the moderators. Player type was coded as 1=recreational player and 2=professional e-sport player. All variables in the regression models were continuous variables, except for player type. Age and gender were added to the models as covariates. Given the high number of moderation analyses in the case of coping styles, Bonferroni correction was applied. More specifically, the significance level (*P*<.05) was divided by the number of tests (n=8 different coping styles). Consequently, a *P* value of .006 was used as an indicator of statistical significance.

## Results

### Descriptive Statistics

Most of the 3476 gamers in the sample were male (n=3133, 90.13%), and their ages ranged from 14 to 58 years. The average age was 23.2 (SD 6.48) years. The years they spent in education was approximately 13.2 years (SD 3.04). The findings indicated that 57.33% (n=1993) were single, 35.62% (n=1238) were in a relationship, 5.78% (n=201) were married, 0.48% (n=17) were divorced, 0.06% (n=2) were widowed, and 0.72% (n=25) did not provide information regarding their marital status. Over half of the gamers in the sample were still studying (n=1981, 56.99%), 57.31% (n=1992) worked part time or full time, and 17.52% (n=609) of the gamers who were still studying in the educational system also worked. On an average day, the participants played video games for 2.6 (SD 1.31) hours per day and 18.2 (SD 9.20) hours per week. Approximately 1 in 20 gamers (n=161, 4.63%) identified as e-sport gamers, based on their e-sport tournament participation (ie, they participated in e-sport tournaments at least 6-11 times in the past year).

### Factor Analyses

First, confirmatory factor analysis was conducted on the sample to test the model fit of the 14-factor structure of the Brief COPE scale. The model had an acceptable fit to the data (*χ*^2^_231_=45551.7; *P*<.001; comparative fit index 0.963; Tucker-Lewis index 0.947; root mean square error of approximation 0.036 [between 0.035 and 0.038]; squared residual 0.03). However, many of the originally proposed factors had low internal consistencies, such as self-distraction (0.55), venting (0.58), behavioral disengagement (0.61), acceptance (0.66), and planning (0.69). Owing to this and following the approach of previous studies, an EFA was performed to identify an alternative factor structure of the Brief COPE. Principal component analysis with Promax rotation was performed. The following items had high cross-loadings and were therefore excluded from further analyses: items 6, 12, 16, 17, 19, and 21. A new EFA was then performed using Promax rotation. The Kaiser-Meyer-Olkin (KMO) index was also calculated to measure sample size adequacy. In this sample, EFA produced a good KMO value (0.74) [45]. Bartlett test of sphericity was χ^2^_253_=31803.7 (*P*<.001), indicating that the correlation structure was adequate for factor analyses. On the basis of the scree plot, the proportion of total variance, the eigenvalue-one criterion, and the interpretability of the factors, an eight-factor solution, appeared to best fit the data, accounting for 72.75% of variance. The results of the EFA analysis are presented in Tables 1-3.

**Table 3:** Correlation matrix of the study’s variables.

Variables			BSI^a^		IGD^b^		Emotional/social support		Active coping		Self-blame/self-distraction		Humor		Substance use		Denial		Religion		Acceptance
**BSI**																					
	*r*	—^c^																			
	*P* value	—																			
**IGD**																					
	*r*	0.41		—																	
	*P* value	<.001		—																	
**Emotional/social support**																					
	*r*	−0.06		−0.04		—															
	*P* value	.001		0.01		—															
**Active coping**																					
	*r*	−0.18		−0.14		0.39		—													
	*P* value	<.001		<.001		<.001		—													
**Self-blame/self-distraction**																					
	*r*	0.60		0.30		0.14		0.03		—											
	*P* value	<.001		<.001		<.001		.06		—											
**Humor**																					
	*r*	−0.01		0.00		0.16		0.22		0.10		—									
	*P* value	.53		.79		<.001		<.001		<.001		—									
**Substance use**																					
	*r*	0.22		0.10		0.10		−0.01		0.24		0.12		—							
	*P* value	<.001		<.001		<.001		.60		<.001		<.001		—							
**Denial**																					
	*r*	0.41		0.24		0.12		0.00		0.40		0.06		0.18		—					
	*P* value	<.001		<.001		<.001		.91		<.001		.001		<.001		—					
**Religion**																					
	*r*	0.10		0.06		0.15		0.11		0.10		0.06		0.07		0.10		—			
	*P* value	<.001		<.001		<.001		<.001		<.001		.001		<.001		<.001		—			
**Acceptance**																					
	*r*	0.15		0.10		0.08		0.11		0.22		0.21		0.08		0.11		0.04		—	
	*P* value	<.001		<.001		<.001		<.001		<.001		<.001		<.001		<.001		.03		—	

^a^BSI: Brief Symptom Inventory.

^b^IGD: internet gaming disorder.

^c^Not applicable.

### The Moderation Models

To investigate the moderating effect of coping strategies on the association between psychiatric symptoms and the symptoms of GD, 8 moderation models were tested. The variable of psychiatric symptoms was entered as the independent variable, GD was the outcome variable, and coping strategies were the moderators. Gender and age were treated as control variables and were added to the models as covariates.

**Figure 1:**
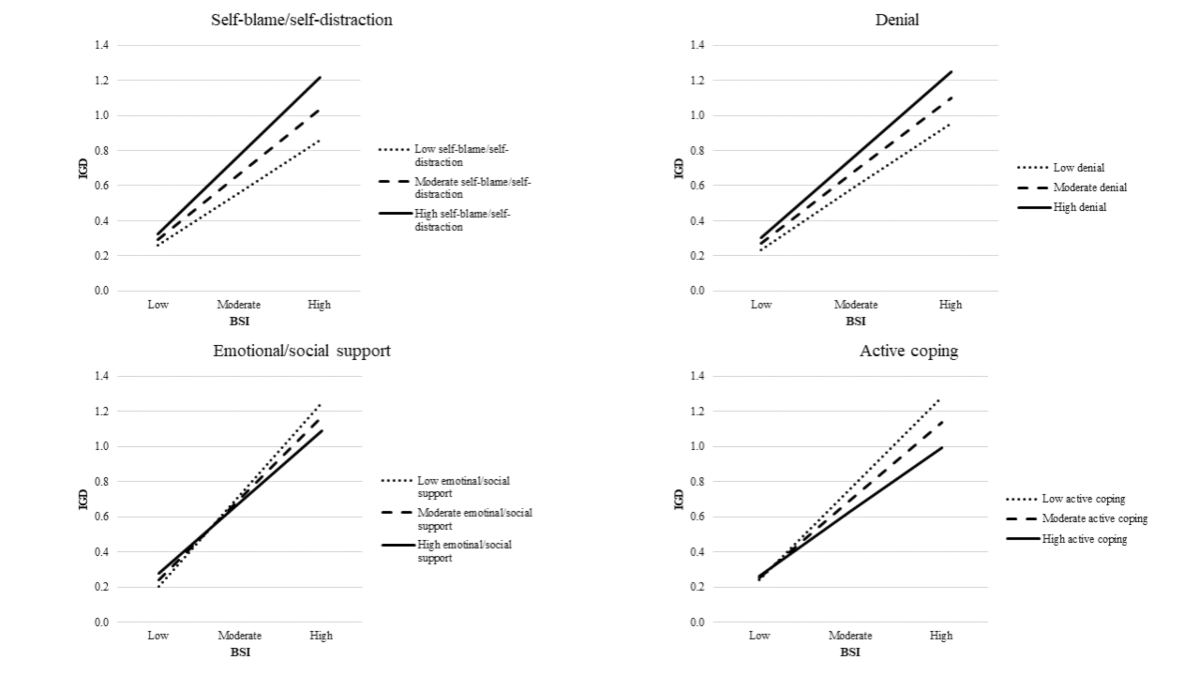
Two-way interaction effect between coping strategies and psychiatric symptoms on gaming disorder. Brief Symptom Inventory represents the scores of Brief Symptom Inventory of psychiatric symptoms. Gaming disorder represents the scores of the 10-Item Internet Gaming Disorder Test. BSI: Brief Symptom Inventory; IGD: internet gaming disorder.

The main effect of psychiatric symptoms was moderate to large (ranging from 0.35 to 0.40) in all 8 models. The interaction terms (ie, moderation effects) were significant for 4 of the 8 coping strategies. However, these did not increase the explained variance of the models considerably (R^2^ change ranged from 0.003 to 0.005 or 0.3% to 0.5% change in the variance; Table 4). More specifically, the moderator effects of self-blame/self-distraction (*β*=.07; *P*<.001) and denial (*β*=.05; *P*=.001) strategies on the association between psychiatric symptoms and the symptoms of GD were significant. When the level of psychiatric symptoms was low, the level of GD symptoms was also low, irrespective of the levels of these coping styles. However, when the level of psychiatric symptoms was high, the level of GD symptoms varied based on the level of coping styles the players applied. Those who use self-blame/self-distraction and denial coping styles more experience significantly more GD symptoms than those who use these coping styles less (Figure 1). Moreover, the moderating effect of emotional/social support (*β*=−.05; *P*=.001) and active coping (*β*=−.06; *P*<.001) on the relationship between psychiatric symptoms and GD was also significant. More specifically, when the level of psychiatric symptoms was low, the level of GD symptoms was also low, irrespective of the levels of these coping styles. However, when the level of psychiatric symptoms was high, the level of GD symptoms varied based on the level of coping styles the players applied. Those who use emotional/social support and active coping more experience significantly less GD symptoms than those who use these coping styles more (Figure 1). However, the moderating effect of coping strategies on the association between psychiatric symptoms and symptoms of GD was generally weak in all models. Furthermore, the moderating effects of the other coping strategies, namely acceptance (*β*=.04; *P*=.02), substance use (*β*=−.04; *P*=.02), humor (*β*=−.02; *P*=.21), and religion (*β*=.00; *P*=.98) were not significant after Bonferroni correction was applied (see the *Statistical Analysis* section).

**Table 4:** Moderation analyses of 8 coping styles on the association between psychiatric symptoms and symptoms of gaming disorder.

Models of moderation analyses		*β*	*P* value	R^2^		R^2^ change due to the interaction		*P* value	
**Model 1**					0.179		0.003		.03
	BSI^a^	.394	<.001						
	Emotional/social support	−.018	.25						
	BSI×emotional/social support	−.052	.001						
**Model 2**					0.184		0.005		.002
	BSI	.377	<.001						
	Active coping	−.056	<.001						
	BSI×active coping	−.071	<.001						
**Model 3**					0.185		0.004		.004
	BSI	.316	<.001						
	Self-blame/self-distraction	.090	<.001						
	BSI×self-blame/self-distraction	.069	<.001						
**Model 4**					0.177		0.000		.40
	BSI	.400	<.001						
	Humor	.003	.82						
	BSI×humor	−.019	.21						
**Model 5**					0.178		0.001		.09
	BSI	.399	<.001						
	Substance use	.030	.07						
	BSI×substance use	−.039	.02						
**Model 6**					0.184		0.003		.02
	BSI	.353	<.001						
	Denial	.076	<.001						
	BSI×denial	.053	.001						
**Model 7**					0.177		0.000		.99
	BSI	.399	<.001						
	Religion	.019	.23						
	BSI×religion	.000	.98						
**Model 8**					0.179		0.001		.11
	BSI	.391	<.001						
	Acceptance	.047	<.001						
	BSI×acceptance	.036	.02						

^a^BSI: Brief Symptom Inventory.

Finally, the moderating role of player type (recreational vs e-sport players) was investigated in the association between psychiatric symptoms and symptoms of GD. The main effect of psychiatric symptoms was also moderate to large (*β*=.39) in this model. Furthermore, although the interaction term was significant (*β*=.04; *P*=.02), the R^2^ change due to the interaction was negligible (0.001% or 0.1% change in the explained variance) and nonsignificant (Table 5). Therefore, the results suggest that e-sport players with more severe psychiatric symptoms are not at a considerably higher risk of encountering symptoms of GD compared to recreational players.

**Table 5:** Moderation analyses of player style on the association between psychiatric symptoms and symptoms of gaming disorder.

Models of moderation analyses		*β*	*P* value	R^2^		R^2^ change due to the interaction		*P* value	
**Model**					0.178		0.001		.11
	BSI^a^	.394	<.001						
	e-sport	.035	.03						
	BSI×e-sport	.038	.02						

^a^BSI: Brief Symptom Inventory.

## Discussion

This study explored the moderating effect of a wide range of coping strategies and player type (recreational vs e-sport players) on the association between psychiatric symptoms and GD. It was assumed that individuals who frequently used putatively maladaptive or dysfunctional coping styles when encountering stressful situations in their lives would have stronger psychiatric symptoms or GD bonds than those who used putatively adaptive coping strategies in general. In addition, it was assumed that e-sport players would not significantly differ from recreational players in their psychiatric symptoms or GD association. According to the results regarding coping strategies, the main effect of psychiatric symptoms was moderate to large in all models, which is in line with previous research findings [46-50]. The interaction terms (ie, moderation effects) were significant for 4 of the 8 coping strategies (ie, self-blame/self-distraction, denial, emotional/social support, and active coping). However, the explained variance of the models only increased negligibly (from 0.3% to 0.5%). The direction of the moderations was as expected (ie, putatively maladaptive strategies were associated with more GD symptoms when the level of psychiatric symptoms was high, whereas putatively adaptive strategies were associated with less).

However, the negligible effect sizes of these moderations make the results more comparable to those reported by Brand et al [29]. They tested whether dysfunctional coping styles, namely denial, substance use, and disengagement, moderate the association between psychopathological aspects (ie, depression and social anxiety) and general internet addiction (including online gaming) but found no considerable moderation effect. In contrast, they found that dysfunctional coping styles mediated between psychopathological aspects and general internet addiction. According to their explanation, higher symptoms of depression and social anxiety can increase the risk of dysfunctional coping strategies, which is associated with higher internet addiction rates. Similarly, many other studies have reported that specific coping styles (eg, avoidance, media-focused coping) mediate between psychiatric symptoms or stress and GD. Given that specific coping styles (especially putatively maladaptive ones as aforementioned) are associated with psychiatric symptoms at moderate or moderate-to-strong levels and with GD at weak or weak-to-moderate levels [26-28] (and in this study; Table 1), it was expected that they would have a mediating effect. A mediating effect suggests that when experiencing high levels of psychiatric symptoms, individuals are more likely to use specific putatively maladaptive coping styles. For instance, in a naturalistic study, a high level of depressive symptoms was associated with an increased use of experiential avoidance on a daily basis [51].

Nevertheless, as mentioned in the *Introduction*, it is also plausible to think that individuals who frequently (ie, habitually) use putatively maladaptive or dysfunctional coping styles when encountering stressful situations in their lives have stronger psychiatric symptoms or GD bonds than those who use putatively adaptive coping strategies in general. However, it has also been suggested that in concrete situations, numerous factors influence the coping or emotion regulation strategy selection applied by an individual [52]. This would explain why no considerable moderation effects were found even if coping strategies were dispositional or trait-like to a certain degree. Overall, additional studies are necessary to confirm these findings, and longitudinal studies and experiments should be designed to explore the causal relationships in the etiology of GD and to better understand these crucial coping-related mechanisms.

Finally, the assumption regarding the effect of player type (recreational vs e-sport players) on the association between psychiatric symptoms and GD was met because the change in explained variance of the moderation model was negligible (0.1%). To date, very few empirical studies have investigated whether e-sport players are at a higher risk of developing GD than recreational gamers. Studies that compared e-sport players and recreational players found significant differences in motivation [34,53] but reported no significant differences in GD and GD-related mechanisms (eg, the mediation effect of gaming motives between psychiatric symptoms and GD; Bányai et al [34]). This is also in line with findings suggesting that increased time spent gaming is not associated with psychiatric problems and is not a good predictor of GD [54]. These results suggest that e-sport players are not necessarily at a higher risk of developing GD than those who are highly engaged recreational gamers. This is also plausible, knowing how goal-oriented and structured e-sport training is [6]. Players have a tight daily schedule, including proper time for eating healthily, sleeping properly, and performing physical exercise. Moreover, they often train in teams and cultivate social bonds while playing as well. Nevertheless, it is important to conduct more research in this field of e-sports and to investigate the risk of GD among e-sport players [33,55].

This study has several limitations that should be noted when interpreting the findings. Although the sample of this study was large, because of its self-selected nature, the results should be generalized with caution. Furthermore, biases of self-report surveys (eg, memory recall and social desirability) should also be considered when interpreting the results. The categorization of e-sports and recreational gamers was based on the number of self-reported gaming competitions they engaged in (ie, frequency of e-sport competitions). Future studies should use more standardized methods for this categorization. Coping strategies were assessed generally and did not consider how individuals cope with different types of stressors. However, it is worth noting that habitually used maladaptive emotion regulation strategies are associated with increased negative affect and atypical cortisol responses to psychosocial stressors in laboratory studies [56]. This suggests that the frequent use of these strategies in different situations may create a vulnerability to mental health problems. Finally, due to the cross-sectional design of the study, causal explanations could not be drawn. Longitudinal and experimental studies should be conducted to address this limitation.

Despite these limitations, this study investigated important questions using a large sample of highly engaged video game players and a subsample of e-sport players. The relationship between psychiatric symptoms and GD has been consistently confirmed. Therefore, understanding factors that attenuate or aggravate this relationship will help in planning better intervention programs. Strategies individuals routinely use to cope with stress and regulate their negative affect can be considered when considering these factors. Understanding why a given individual uses specific affect regulation strategies in a given situation or across situations and the emotional or behavioral consequences of the ways of affect regulation [57] could be an essential component of personalized treatments targeting mental health problems including GD. Furthermore, in relation to prevention and intervention programs, experts should focus on both the coping strategies of the individuals and their style of video game usage. Playing video games can be viewed as a coping strategy. As a media-focused coping style, video game playing could have similar outcomes as self-distraction and behavioral disengagement among some problematic gamers or players diagnosed with GD [22,58,59] when playing games to avoid aversive and stressful situations [60]. Gaming is a recreational activity that primarily serves pleasure, relaxation, and/or stress-relief. However, gamers can also avoid discomfort and escape from their problems in real life. In this regard, future research should focus on the style of video game playing and how this activity is integrated into gamers’ lives (eg, recreation, e-sport or problematic gaming, or an escape option from reality). Individual coping styles and emotion regulation strategies also play a role in this and therefore should be considered in the prevention and treatment processes.

## Abbreviations

BSI: Brief Symptom Inventory
EFA: exploratory factor analysis
GD: gaming disorder
IGD: internet gaming disorder
IGDT-10: 10-Item Internet Gaming Disorder Test
KMO: Kaiser-Meyer-Olkin
